# Minimally Invasive Mitral Valve Repair for Commissural Prolapse: Safety, Success, and Long-Term Efficacy

**DOI:** 10.1093/icvts/ivaf213

**Published:** 2025-09-27

**Authors:** Clemens Engler, Leo Pölzl, Felix Nägele, Michael Graber, Jakob Hirsch, Ronja Lohmann, Johannes Holfeld, Julia Dumfarth, Johannes Spilka, Ludwig Müller, Michael Grimm, Daniel Höfer, Can Gollmann-Tepeköylü, Nikolaos Bonaros

**Affiliations:** Department of Cardiac Surgery, Medical University of Innsbruck, Innsbruck 6020, Austria; Department of Cardiac Surgery, Medical University of Innsbruck, Innsbruck 6020, Austria; Department of Cardiac Surgery, Medical University of Innsbruck, Innsbruck 6020, Austria; Department of Cardiac Surgery, Medical University of Innsbruck, Innsbruck 6020, Austria; Department of Cardiac Surgery, Medical University of Innsbruck, Innsbruck 6020, Austria; Department of Cardiac Surgery, Medical University of Innsbruck, Innsbruck 6020, Austria; Department of Cardiac Surgery, Medical University of Innsbruck, Innsbruck 6020, Austria; Department of Cardiovascular Surgery, German Heart Center Munich, School of Medicine & Health, Technical University of Munich, Munich 80333, Germany; Department of Cardiac Surgery, Medical University of Innsbruck, Innsbruck 6020, Austria; Department of Cardiac Surgery, Medical University of Innsbruck, Innsbruck 6020, Austria; Department of Cardiac Surgery, Medical University of Innsbruck, Innsbruck 6020, Austria; Department of Cardiac Surgery, Medical University of Innsbruck, Innsbruck 6020, Austria; Department of Cardiac Surgery, Medical University of Innsbruck, Innsbruck 6020, Austria; Department of Cardiac Surgery, Medical University of Innsbruck, Innsbruck 6020, Austria; Department of Cardiac Surgery, Medical University of Innsbruck, Innsbruck 6020, Austria

**Keywords:** commissural prolapse, minimally invasive mitral valve repair, mitral valve surgery, Barlow’s disease

## Abstract

**Objectives:**

Commissural prolapse (CP) is a rare and complex mitral valve pathology which is complicated in preoperative diagnosis and repair. This study evaluated the safety, success, and long-term efficacy of minimally invasive mitral valve repair (MIMVR) for CP compared to posterior leaflet prolapse (PMLp).

**Methods:**

Between 2001 and 2022, 34 patients with CP and 590 with PMLp underwent MIMVR at our centre. Operative, perioperative, and long-term follow-up data were retrospectively collected. Surgical success was defined as freedom from conversion to valve replacement, sternotomy, and residual mitral regurgitation (MR) > grade I. Long-term efficacy included MR recurrence, reoperation, and survival.

**Results:**

Median age was similar (CP: 64.0 years [53.2; 69.8] vs PMLp: 62.0 years [53.0; 69.0], *P* = .783). Barlow’s disease was more frequent in PMLp (80.3% vs 32.4%, *P* < .001). Cross-clamp (127.0 min vs 105.0 min, *P* = .001) and bypass times (208.5 min vs 190.5 min, *P* = .031) were longer in CP. CP patients had longer hospital stays (10.0 days [8.0; 12.0] vs 8.0 days [7.0; 9.0], *P* < .001), but short-term outcomes, including 30-day mortality, stroke, and extracorporeal membrane oxygenation support, were comparable. At a median follow-up of 4.5 years (CP) and 2.4 years (PMLp, *P* = .001), rates of recurrent MR, reoperation, and survival were similar.

**Conclusions:**

MIMVR for CP is safe, successful, and durable with outcomes comparable to PMLp. Despite greater technical complexity and longer operative times, this approach provides reliable results for CP.

## Graphical abstract

**Figure:**
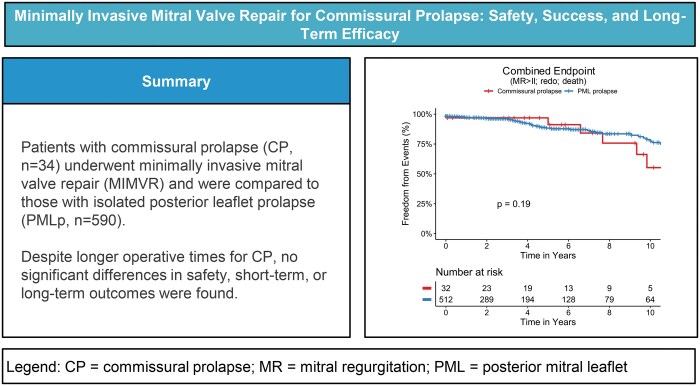

## INTRODUCTION

Barlow’s disease is a primary degenerative mitral valve disorder in which myxoid infiltration of the valve results in excess leaflet tissue, different phenotypes of chordal anatomy ranging from normal to elongated or ruptured chords and bileaflet prolapse.^1–3^ These structural abnormalities often result in significant mitral regurgitation (MR), posing both diagnostic and surgical challenges.^4^ Commissural prolapse (CP), a distinct and technically demanding subset of mitral valve pathology, involves the prolapse of the commissures—where the anterior and posterior leaflets converge forming an anatomical and sometimes functional commissural segment.^5^ This condition is anatomically complex and requires precise restoration of leaflet coaptation to achieve durable repair. Posteromedial CP is more commonly observed than anterolateral prolapse, highlighting the variability within this pathology.^4^^,^^6–8^

Surgical repair of CP often requires advanced techniques tailored to the complex commissural anatomy. Conventional approaches like midline sternotomy or hemisternotomy are well-studied. Kim et al highlighted the technical challenges, including longer operative times, while Shimizu et al emphasized the need for individualized strategies to achieve durable outcomes in complex mitral valve cases.^6^^,^^7^ Despite these advances, limited evidence exists regarding the role of minimally invasive mitral valve repair (MIMVR) in addressing CP.

CP is not only difficult to diagnose but also inherently challenging to repair due to the complex anatomical relationships and the need for precise leaflet alignment.^4^^,^^6–8^ Compared to more common mitral pathologies, such as posterior mitral leaflet prolapse (PMLp), commissural repairs are often associated with prolonged cross-clamp times and require advanced surgical expertise.^4^^,^^7–17^ Moreover, the clinical characteristics of patients with CP, including demographic and echocardiographic profiles, remain underreported. A key question is whether MIMVR, as an advanced surgical approach, can overcome these technical challenges and deliver outcomes comparable to or better than conventional methods.

This study aims to evaluate the feasibility, safety, and long-term efficacy of MIMVR in patients with CP. Specifically, it investigates whether MIMVR can achieve favourable perioperative and long-term outcomes in this complex mitral valve pathology. Addressing this knowledge gap may offer insights into MIMVR as a viable and reliable treatment strategy for CP.

## METHODS

### Patients

This study included consecutive patients undergoing MIMVR at the Medical University of Innsbruck between 2001 and 2022. Surgical decisions were made by the heart team. All procedures were performed via anterolateral thoracotomy with 2D or 3D endoscopy. CP was diagnosed by an experienced echocardiographer and confirmed intraoperatively. Follow-up was obtained through chart review, in-house evaluations, telephone interviews, and electronic records; mortality data came from Statistik Austria. The study was approved by the Ethics Committee (1203/2019) and conducted in accordance with national regulations and the Declaration of Helsinki.

### Definitions of outcomes

Operative success was defined as successful mitral valve repair without conversion to replacement, no larger thoracic incisions, no more than mild residual MR, and no reoperation within 30 days. Perioperative safety required absence of mortality, myocardial infarction (per Fourth Universal Definition), stroke, extracorporeal membrane oxygenation (ECMO) support, or reoperation for bleeding within 30 days.^18^

Reoperation-free survival, a key indicator of long-term efficacy, was defined as freedom from death or reoperation during follow-up. Valve-related complications assessed retrospectively included residual MR, prosthetic valve dysfunction (paravalvular leak, degeneration, thrombosis), and mitral valve endocarditis.^16^^,^^19^^,^^20^

### Surgical technique

#### Intraoperative decision-making for CP repair

Decision criteria for artificial chordae, resection, or sliding plasty in CP resemble those for classic PMLp but differ in some aspects. Artificial chordae are preferred with extensive prolapse, suitable papillary muscle anatomy and tissue quality (avoiding small intermediate papillary muscles), and no excessive tissue. Commissural resection is chosen for limited prolapse or adjacent scallop involvement in patients with excessive tissue (eg, Barlow’s disease). The use of sliding plasty requires extensive prolapse of the commissure in addition to adjacent segments at the presence of excessive tissue. The selection of the technique used was left at the discretion of the operating surgeon and was based on the intraoperative findings of meticulous valve analysis (Video S1) (**Table 5**).

Anatomical treatment of CP was performed by one of the following techniques. Except for endocarditis, all patients underwent subsequent mitral valve annuloplasty.

#### Leaflet resection

Resection of adjacent leaflets scallops has been used in patients with concomitant prolapse of the pre-commissural area. This pathology can be seen at the P3 area with involvement of the posteromedial commissure. A small triangular leaflet resection together with resuspension of the commissural tissue is usually sufficient to address the prolapse or flail. Additional commissural single stiches were rarely used to support the commissure.

#### Extended leaflet sliding

In extended CP with involvement of both adjacent segments of the peri-commissural tissue, resection of the commissural segment with simultaneous sliding plasty of the neighbouring scallops of the posterior and the anterior leaflet was performed using a continuous 5/0 polypropylene suture.

#### Artificial chordae

In extensive commissural flail pathology, artificial chordae were secured at the corresponding papillary muscle and the prolapsing commissural segment. In this case, the anatomy of the remaining native chords was identified, and the artificial chordae were placed at the same papillary muscle head respecting the anatomical origin of the ruptured chords. Either single ePTFE (GORE-TEX W.L. Gore & Associates Inc., Flagstaff AZ) or PTFE loops (Seramon SERAG-WIESSNER, Nala Germany) were used to replace ruptured or elongated primary chords (Video S1).

#### Commissural plasty

At the beginning of our minimally invasive commissural repair experience, 1 or 2 polypropylene stitches were used to restore commissural coaptation, as in Carpentier’s technique, with additional annular plication stitches if needed.

### Surgical outcomes

All patients in the study underwent MIMVR either video-assisted or totally endoscopic, via an anterolateral mini-thoracotomy. To investigate surgical outcomes, patients with mitral CP were compared to those with PMLp. Comparisons adhered to the Mitral Valve Academic Research Consortium criteria.^16^^,^^19^

### Missing data

Due to the retrospective nature of the study, follow-up was not complete for all patients. Missing data are indicated either in the text or in the total number of patients presented in **Table 4**. Outcome analyses were performed only in patients with complete follow-up data.^21^

### Era analysis

To account for potential temporal confounding, the cohort was divided into 2 eras (2001-2011 and 2012-2022), and overall survival was compared between eras using Kaplan-Meier analysis.

### Statistical analysis

Data distribution was assessed with histograms, box plots, skewness, kurtosis, and the Shapiro-Wilk test. Continuous variables are shown as mean ± SD or median [IQR]. Comparisons used Student’s *t*-test for parametric and Mann-Whitney *U*-test for non-parametric data. A value of *P* < .05 was considered significant. Statistical analyses were conducted using R-Studio (R Core Team, 2023) with the compareGroups package.^22^

Cumulative incidence functions (CIF) were calculated to account for competing events, with death, reoperation, and residual mitral regurgitation > grade II defined as mutually exclusive outcomes. The Fine-Gray subdistribution hazard model was used to compare event incidence between groups, and Gray’s test was applied for hypothesis testing. Follow-up was truncated at 10 years to ensure comparable observation time across groups.

## RESULTS

Between September 2001 and November 2022, a total of 1116 patients underwent MIMVR at our centre. Of these, 34 patients (3.0%) with CP and 590 patients (52.9%) with PMLp were included for analysis of surgical outcomes.

The median age was 64.0 years [IQR 53.2; 69.8] in the CP group versus 62.0 years [IQR 53.0; 69.0] in the PMLp group (*P* > .05) **(Table 1)**. Female patients accounted for 32.4% in the CP group and 30% in the PMLp group (*P* > .05). EuroSCORE II was similar, with a median of 1.3% [0.9; 1.6] in the CP group and 1.2% [0.8; 2.0] in the PMLp group (*P* > .05).

**Table 1.** Preoperative Characteristics

	Commissure	PML	
	n = 34	n = 590	*P*-value
Age (years), median [IQR]	64.0 [53.2; 69.8]	62.0 [53.0; 69.0]	.783
Female, n (%)	11 (32.4)	175 (29.7)	.888
EuroSCORE II (%), median [IQR]	1.3 [0.9; 1.6]	1.2 [0.8; 2.0]	.985
Body mass index (kg/m²), median [IQR]	24.3 [21.9; 26.7]	24.6 [22.5; 27.1]	.421
LVEF (%), median [IQR]	60.0 [54.2; 65.0]	60.0 [55.0; 65.0]	.421
Hypertension, n (%)	21 (61.8)	276 (46.8)	.127
Diabetes, n (%)	3 (8.8)	19 (3.2)	.112
Dyslipidaemia, n (%)	10 (29.4)	187 (31.7)	.929
Smoking, n (%)	4 (11.8)	49 (8.3)	.520
Chronic lung disease, n (%)	1 (2.9)	19(3.2)	1.000
Creatinine, median [IQR]	0.9 [0.8; 1.1]	0.9 [0.8; 1.1]	.909
Dialysis, n (%)	2 (5.9)	2 (0.3)	.016
Peripheral artery disease, n (%)	0 (0.0)	12 (2.0)	1.000
Cerebrovascular arteriopathy, n (%)	1 (2.9)	7 (1.2)	.363
Previous stroke, n (%)	2 (5.9)	8 (1.4)	0.099
Previous PCI, n (%)	0 (0.0)	7 (1.2)	1.000
Recent myocardial infarction, n (%)	0 (0.0)	1 (0.2)	1.000
NYHA			0.008
Class I—n (%)	6 (17.6)	99 (17.2)	
Class II—n (%)	10 (29.4)	249 (43.3)	
Class III—n (%)	15 (44.1)	223 (38.8)	
Class IV—n (%)	3 (8.8)	4 (0.7)	
Atrial fibrillation, n (%)	13 (38.2)	165 (28.0)	0.274
Tricuspid regurgitation			0.429
No regurgitation, n (%)	16 (47.1)	204 (34.7)	
Grade I, n (%)	2 (5.9)	52 (8.8)	
Grade II, n (%)	16 (47.1)	330 (56.1)	
Grade III, n (%)	0 (0.0)	2 (0.3)	
Mitral regurgitation			0.177
Grade I, n (%)	1 (2.9)	2 (0.3)	
Grade II, n (%)	0 (0.0)	5 (0.8)	
Grade III, n (%)	33 (97.1)	583 (98.8)	
Mitral disease aetiology			0.340
Degenerative, n (%)	32 (94.1)	556 (94.2)	
Functional, n (%)	1 (2.9)	29 (4.9)	
Rheumatic, n (%)	0 (0.0)	2 (0.3)	
Endocarditis, n (%)	1 (2.9)	3 (0.5)	
Degenerative type			<0.001
No degeneration	5 (14.7)	17 (2.9)	
Mb. Barlow, n (%)	11 (32.4)	473 (80.3)	
Fibroelastic deficiency, n (%)	18 (52.9)	99 (16.8)	
Prolapse			<0.001
No prolapse	2 (5.9)	0 (0.0)	
AML, n (%)	3 (8.8)	0 (0.0)	
PML, n (%)	12 (35.3)	590 (100.0)	
AML + PML, n (%)	17 (50.0)	0 (0.0)	
Carpentier classification			0.547
Type I, n (%)	1 (2.9)	7 (1.2)	
Type II, n (%)	33 (97.1)	577 (97.8)	
Type IIIa, n (%)	0 (0.0)	5 (0.8)	
Type IIIb, n (%)	0 (0.0)	1 (0.2)	

Abbreviations: AML, anterior mitral leaflet; EuroSCORE II, The European System for Cardiac Operative Risk Evaluation II; LVEF, left ventricular ejection fraction; NYHA, New York Heart Association; PCI, percutaneous intervention; PML, posterior mitral leaflet.

Left ventricular ejection fraction (LVEF) was the same in the CP group (60% [54; 65]) compared to the PMLp group (60% [55; 65], *P* > .05). Barlow disease was significantly more prevalent in the PMLp group (80.3% vs 32.4%, *P* < .001).

### Perioperative data

The operative details of both groups is summarized in **Table 2**. Artificial chord implantation differed significantly between groups (*P* = .017). In the CP group, artificial chords were more frequently placed on the adjacent anterior mitral leaflet (AML: 27.3% vs 9.7%), while in the PMLp group, chords were predominantly placed on the adjacent posterior mitral leaflet (PML: 30.3% vs 46.2%). Combined AML and PML chord implantation were rare in both groups but occurred less frequently in CP patients (3.0% vs 7.7%).

**Table 2.** Operative Details

	Commissure	PML	
	n = 34	n = 590	*P*-value
Artificial chords			.017
AML, n (%)	9 (27.3)	57 (9.7)	
PML, n (%)	10 (30.3)	270 (46.2)	
AML + PML, n (%)	1 (3.0)	45 (7.7)	
Resection			.008
AML, n (%)	0 (0.0)	2 (0.3)	
PML, n (%)	7 (20.6)	198 (33.7)	
AML + PML, n (%)	2 (5.9)	1 (0.2)	
Sliding plasty, n (%)	1 (2.9)	23 (4.0)	1.000
Annuloplasty ring, n (%)	33 (97.1)	580 (98.3)	.463
Ring diameter (mm), median [IQR]	34.0 [30.0; 36.0]	34.0 [32.0; 36.0]	.739
Conversion to valve replacement, n (%)	1 (2.9)	8 (1.4)	.398
Conversion to sternotomy, n (%)	2 (5.9)	11 (1.9)	.183

Abbreviations: AML, anterior mitral leaflet; PML, posterior mitral leaflet.

Leaflet resection also showed significant differences between groups (*P* = .008). In the CP group, PML resections occurred in 20.6% of cases compared to 33.7% in the PMLp group, while combined AML and PML resections were more common in the CP group (5.9% vs 0.2%). Sliding plasty was performed in 2.9% of CP cases and 4.0% of PMLp cases, with no significant difference between groups (*P* = 1.000).

Annuloplasty rings were used in nearly all patients (97.1% in CP vs 98.3% in PML, *P* = .463), and the median ring diameter was similar between groups (34.0 mm [30.0; 36.0] in CP vs 34.0 mm [32.0; 36.0] in PMLp, *P* = .739). Conversion rates to valve replacement or sternotomy were low overall, with no statistically significant differences observed (valve replacement: 2.9% vs 1.4%, *p* = 0.398; sternotomy: 5.9% vs 1.9%, *P* = .183).

### Outcomes

Cardiopulmonary bypass time was longer in the CP group, with a median of 208.5 min [IQR 184.8; 238.2] compared to 190.5 min [IQR 156.0; 232.0] in the PMLp group (*P* = 0.031) **(Table 3**). Similarly, cross-clamp time was notably higher in the CP group (127.0 min [IQR 112.2; 145.5]) versus 105.0 min [IQR 83.0; 126.0] in the PMLp group (*P* = .001).

**Table 3.** Peri- and Postoperative Data

	Commissure	PML	
	n = 34	n = 590	*P*-value
Bypass time in min, median [IQR]	208.5 [184.8; 238.2]	190.5 [156.0; 232.0]	.031
X-Clamp in min, median [IQR]	127.0 [112.2; 145.5]	105.0 [83.0; 126.0]	.001
Repeated clamping, n (%)^a^	0 (0.0)	23 (3.9)	.629
Mitral regurgitation			.915
No regurgitation, n (%)	25 (73.5)	432 (73.2)	
Grade I, n (%)	9 (26.5)	147 (24.9)	
Grade II, n (%)	0 (0.0)	11 (1.9)	
Intubation time (hours), median [IQR]	9.5 [6.2; 15.8]	8.0 [5.0; 12.0]	.154
Prolonged ventilation, n (%)^b^	2 (5.9)	48 (8.1)	1.000
Reintubation or tracheostomy, n (%)	0 (0.0)	18 (3.1)	.616
Intensive care unit stay in hours, median [IQR]	19.0 [11.5; 20.0]	20.0 [19.0; 22.0]	.001
Extracorporeal membrane oxygenation, n (%)	0 (0.0)	31 (5.3)	.403
Bleeding revision, n (%)	2 (5.9)	28 (4.7)	.675
Redo for early failure, n (%)	0 (0.0)	5 (0.08)	1.000
Atrial fibrillation, n (%)	5 (14.7)	154 (26.1)	.212
Stroke, n (%)	0 (0.0)	1 (0.2)	.637
Myocardial infarction, n (%)	0 (0.0)	2 (0.3)	.657
Hospital stay (days), median [IQR]	10.0 [8.0; 12.0]	8.0 [7.0; 9.0]	<.001
30-day mortality, n (%)	1 (2.9)	3 (0.5)	.202
In-hospital mortality, n (%)	0 (0.0)	6 (1.0)	1.000

a Repeated clamping due to residual mitral regurgitation

b Ventilation for more than 24 hours.

Postoperative intensive care unit (ICU) stay was slightly shorter in the CP group, with a median of 19.0 hours [IQR 11.5; 20.0] compared to 20.0 hours [IQR 19.0; 22.0] in the PMLp group (*P* = .001). The overall hospital stay was significantly longer for CP patients (10.0 days [IQR 8.0; 12.0]) compared to the PMLp group (8.0 days [IQR 7.0; 9.0], *P* < .001). Other perioperative outcomes, including rates of residual MR, reintubation, prolonged ventilation, bleeding revisions, and atrial fibrillation, were similar between the two groups. Repeated clamping was not required in the CP group but occurred in 3.9% of the PMLp group (*P* = .629). The incidence of complications, such as stroke, myocardial infarction, or ECMO support, was low and comparable between groups. Thirty-day mortality was 2.9% in the CP group and 0.5% in the PMLp group (*P* = .202). The median follow-up duration was 46.5 months [23.5; 62.2] in the CP and 33.0 months [18.0; 50.0] in PMLp (*P* = .039). 53.0% (*n* = 18/34) of CP patients completed the follow-up and 43.6% (*n* = 257/590) of PMLp patients (**Table 4**).

**Table 4.** Follow-up

	Commissure	PML	
	*N = 18*	*N = 257*	
Follow-up (months), median [IQR]	46.5 [23.5; 62.2]	33.0 [18.0; 50.0]	0.039
Residual mitral regurgitation			0.815
No regurgitation, n (%)	7 (38.9)	96 (37.4)	
Grade I, n (%)	9 (50.0)	130 (50.6)	
Grade II, n (%)	1 (5.6)	23 (8.9)	
Grade III, n (%)	1 (5.6)	8 (3.1)	
LVEF, median [IQR]	56.5 [52.3; 60.0]	60.0 [55.0; 60.0]	0.373
Reoperation, n (%)	1 (5.6)	12 (4.7)	0.594

Abbreviation: LVEF, left ventricular ejection fraction.

**Table 5.** Decision Making for Repair in Commissural Prolapse

**Limited commissural prolapse** (Definition: Single leaflet involvement, Narrow prolapsing segment)	Triangular resection Commissural plication
**Extensive pure commissural prolapse** (Definition: Wide prolapsing segment, Single or bi-leaflet pathology)	Artificial chordae or loops on the commissural area Commissural plasty (“magic stitch”)
**Extensive commissural prolapse in addition to prolapse of adjacent segments** (Definition Bileaflet pathology, Additional segments involved (eg, A2/3, P2/3), Fibrotic or calcified areas)	Artificial chordae or loops on the commissural area and the adjacent segments of the anterior and posterior leaflet Resection of the prolapsing adjacent scallop with resuspension of the commissural segment Sliding leaflet plasty of both the anterior and the posterior leaflet (rotation commissural plasty)

To assess temporal confounding, the cohort was split into two 10-year eras and overall survival compared, no significant difference was found (**Figure S4**).

**Figure 1.**
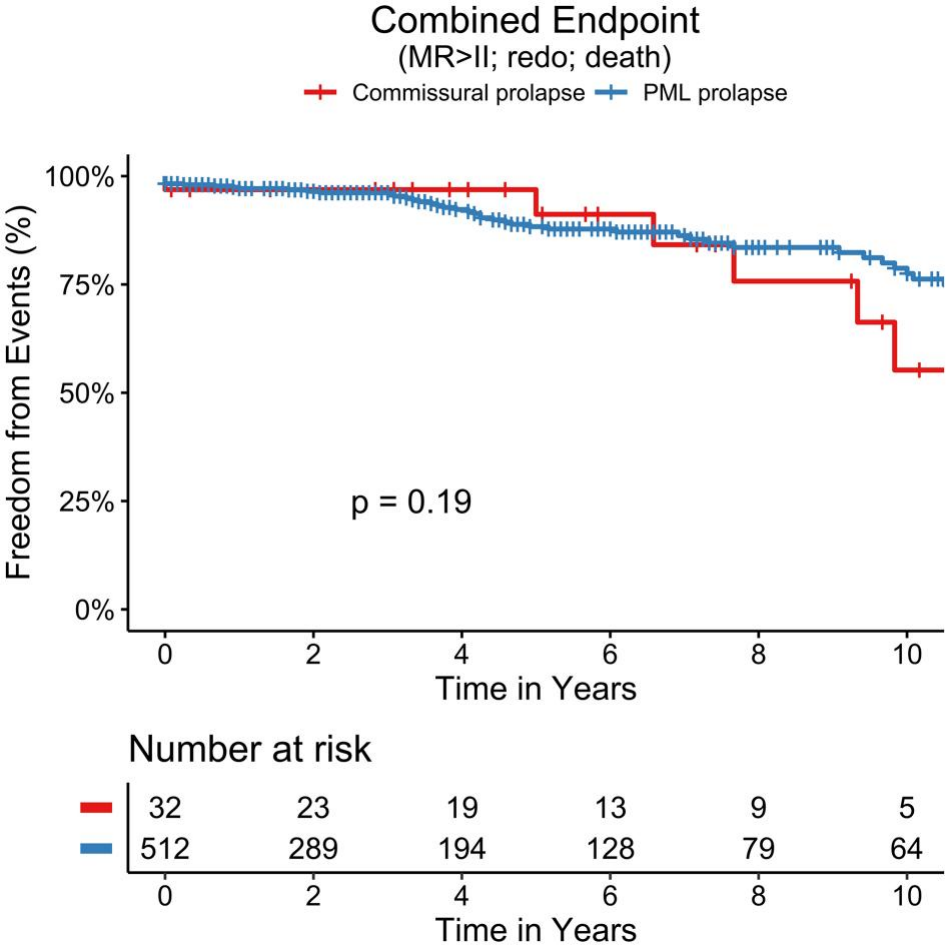
Kaplan-Meier estimates show no difference in the freedom from the combined endpoint between commissural and posterior leaflet prolapse after minimally invasive repair

Rates of recurrent MR were comparable between groups, with no significant differences observed for residual MR grade I-III (**Table 4**, **Figure 1**, **Figures S1 and S3**). Similarly, there were no differences regarding the incidence of valve-related complications, including endocarditis, paravalvular leaks, leaflet degeneration, or device thrombosis. Rates of mitral valve reoperation were low and did not differ significantly between groups. Survival outcomes were also comparable, with 31.2% of CP patients and 19.6% of PMLp patients deceased during the follow-up period. Importantly, both groups demonstrated similar rates of successful mitral valve repair, with no significant differences in long-term outcomes (**Figures 1 and**  **2**, **Figures S1-S3**).

**Figure 2.**
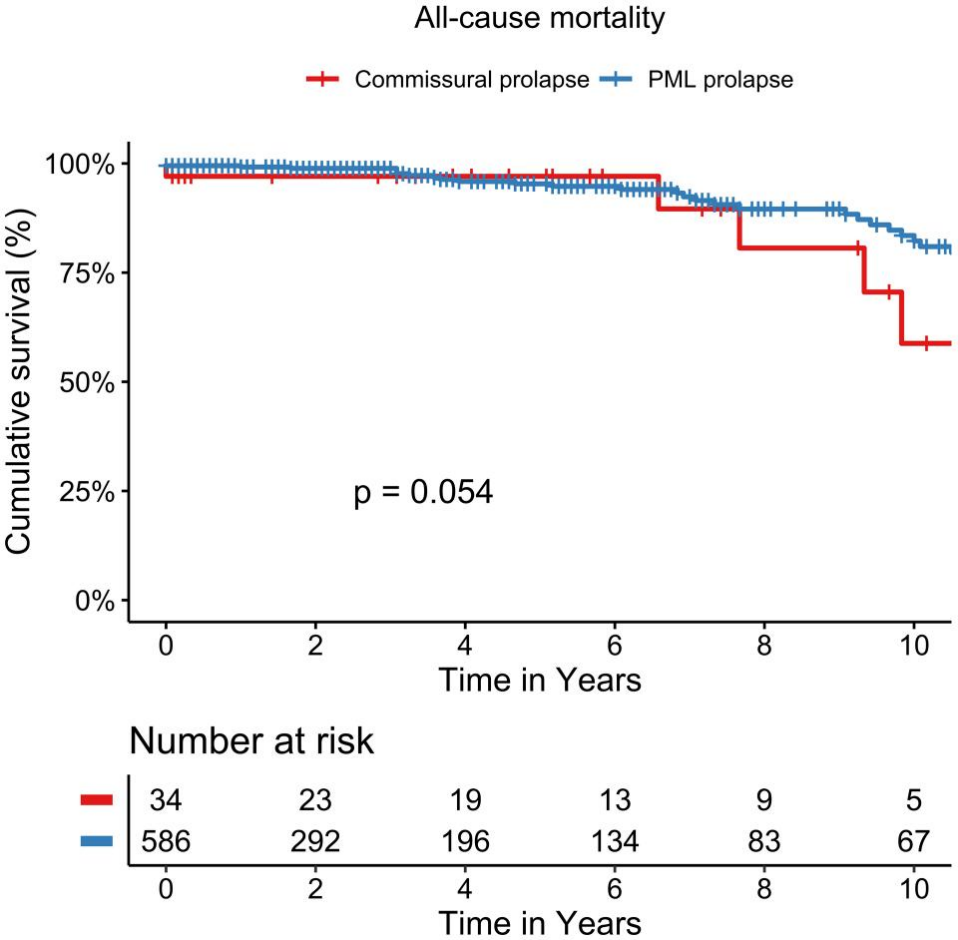
Kaplan–Meier analysis showed no difference in all-cause mortality between commissural and posterior leaflet prolapse after minimally invasive repair

Operative success was achieved in 91.2% of CP patients and 94.7% of those with PMLp (*P* = .422). Perioperative safety was present in 91.2% of CP patients and 90.3% of those with PMLp (*P* = 1.000).

## DISCUSSION

In this study, we evaluated the outcomes of MIMVR in patients with CP and compared these results to the well-established repair of PMLp. The key findings of our analysis are as follows:

1. MIMVR for CP can be performed safely and effectively by experienced surgeons, with short- and long-term outcomes that appear comparable to those of PMLp repair.
2. Although the CP group showed longer cross-clamp times and hospital stays, surgical success rates and durability of repair were comparable.
3. The complex pathology of CP may require versatile repair techniques, such as chordal loops, leaflet resection, and annuloplasty, which yielded favourable results in this single-centre experience.

CP remains a rare and challenging pathology to address, accounting for only 3.4% of our 20-year MIMVR experience. This observation is consistent with the existing literature, which underscores the low incidence of CP across surgical cohorts.^9^ Preoperative diagnosis of CP is inherently difficult due to its complex anatomical presentation and frequent association with leaflet flail or additional prolapse.^4^ Accurate diagnosis requires meticulous imaging and intraoperative confirmation, underscoring the importance of an experienced surgical team and advanced imaging protocols.

Our findings highlight that MIMVR for CP achieves surgical success rates similar to PMLp repair. Specifically, no significant differences were observed in key safety end-points, including 30-day mortality, need for ECMO support, stroke, myocardial infarction, and reoperation for bleeding. This is in line with prior studies evaluating commissural repair in open surgery, which have reported similarly favourable outcomes when performed by experienced surgeons.^7^^,^^10^

The success of MIMVR for CP in our cohort reflects the versatility of techniques used. Loops were most common, followed by leaflet resection and annuloplasty, with nearly all patients receiving a prosthetic ring. The median ring size of 34 mm underscores the routine focus on annular stabilization, a key factor for long-term durability. These findings align with existing evidence highlighting its importance in complex mitral repair.

Notably, the CP group experienced longer cross-clamp times and hospital stays This observation likely reflects the greater technical complexity of CP repair. The need to “untangle” commissural pathology and its frequent association with adjacent leaflet prolapse prolongs operative times. This imposes a greater risk of myocardial damage. All procedures were performed using Custodiol for myocardial protection. Custodiol was administered as a single-dose cardioplegia, given its established efficacy in providing prolonged myocardial protection during extended cross-clamp times.^23^ This did not translate into inferior outcomes, as rates of recurrent MR, endocarditis, valve degeneration, device thrombosis, and mitral valve reoperation during follow-up remained comparable between the groups.

The long-term durability of MIMVR in the CP group was further demonstrated by the absence of significant differences in residual MR and reoperation rates compared to PMLp patients. While survival rates were also similar, the slightly higher mortality observed in the CP group (31.2% vs 19.6%) may reflect the longer median follow-up duration for this cohort (4.5 vs 2.4 years, *P* = .001).This finding highlights the durability of MIMVR in addressing CP, as no increased structural failure or need for reoperation was observed over time.

Although the 30-day mortality in CP patients was not statistically significant, it nevertheless warrants further investigation. Due to the limited number of events, additional subgroup analyses are not feasible within our cohort. To gain more insight into this topic, a prospective international multicentre trial would be necessary, given the rarity of the condition.

Our study adds to the growing evidence supporting the feasibility and reliability of MIMVR in complex pathologies. Compared to open-heart surgery, outcomes were equivalent in safety, efficacy, and durability,^7–9^ though comparisons are limited by heterogeneity in outcome definitions and follow-up duration.

### Limitations

This study has several limitations, including its retrospective design and the low incidence of CP, requiring data collection over a long period. The small number of posterior CP cases limits subgroup analyses, reduces robustness of estimates, and hampers adjustment for confounders. The lack of a contemporary open repair cohort further limits comparisons. Challenges in preoperative imaging and diagnosis may have introduced selection bias. Validation requires prospective, multicentre studies and, ideally, a mitral valve registry with standardized echocardiographic follow-up.

## CONCLUSION

In conclusion, MIMVR for CP can be performed safely and effectively by experienced surgeons. Despite the technical challenges associated with CP repair, outcomes in our cohort appeared comparable to those of PMLp repair, with similar rates of surgical success, safety, and long-term durability. These findings suggest that the minimally invasive approach can be a feasible treatment option for this rare and complex pathology when performed in specialized centres.

## Supplementary Material

ivaf213_Supplementary_Data

## Data Availability

Data underlying will be shared on reasonable request.
