# Supplementary figures and images for: Minimally Invasive Mitral Valve Repair for Commissural Prolapse: Safety, Success, and Long-Term Efficacy
Source: Interdiscip Cardiovasc Thorac Surg. 2025 Sep 27;40(10):ivaf213. doi: 10.1093/icvts/ivaf213 (PMC12526119; doi:10.1093/icvts/ivaf213)

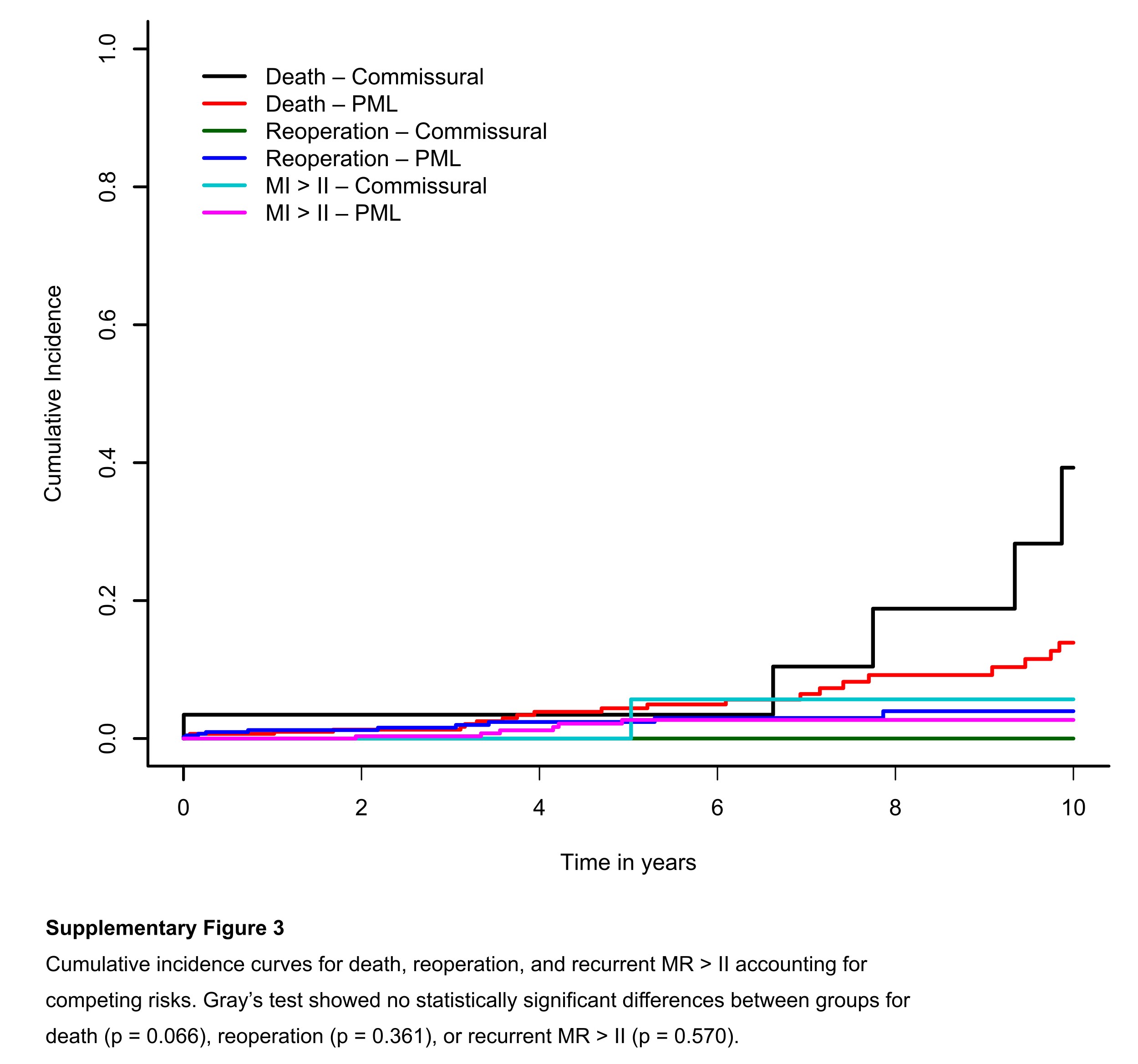

Supplement: ivaf213_Supplementary_Data [file ivaf213_supplementary_data.zip › Supplementary Figure 3.jpg]

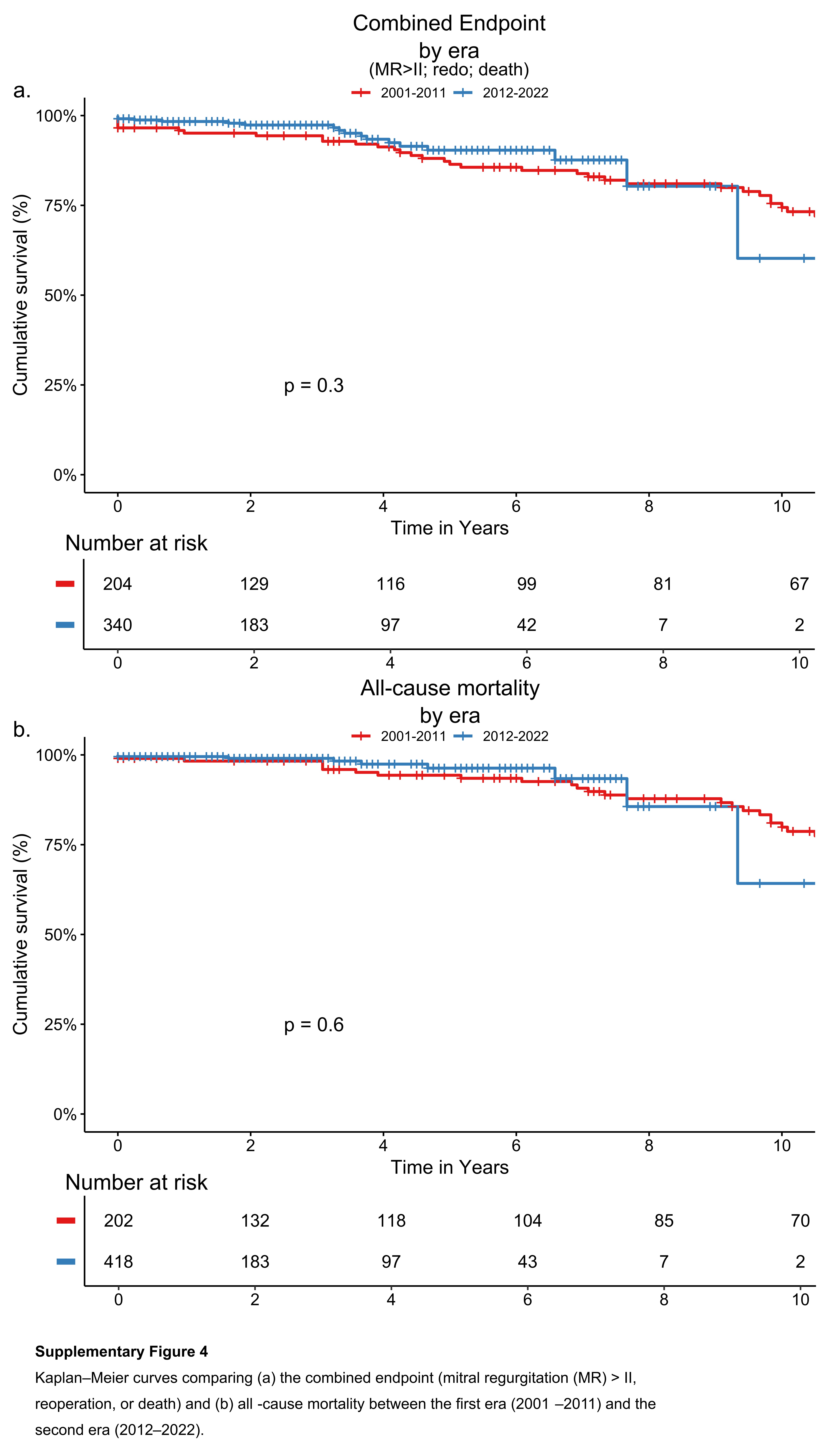

Supplement: ivaf213_Supplementary_Data [file ivaf213_supplementary_data.zip › Supplementary Figure 4.jpg]

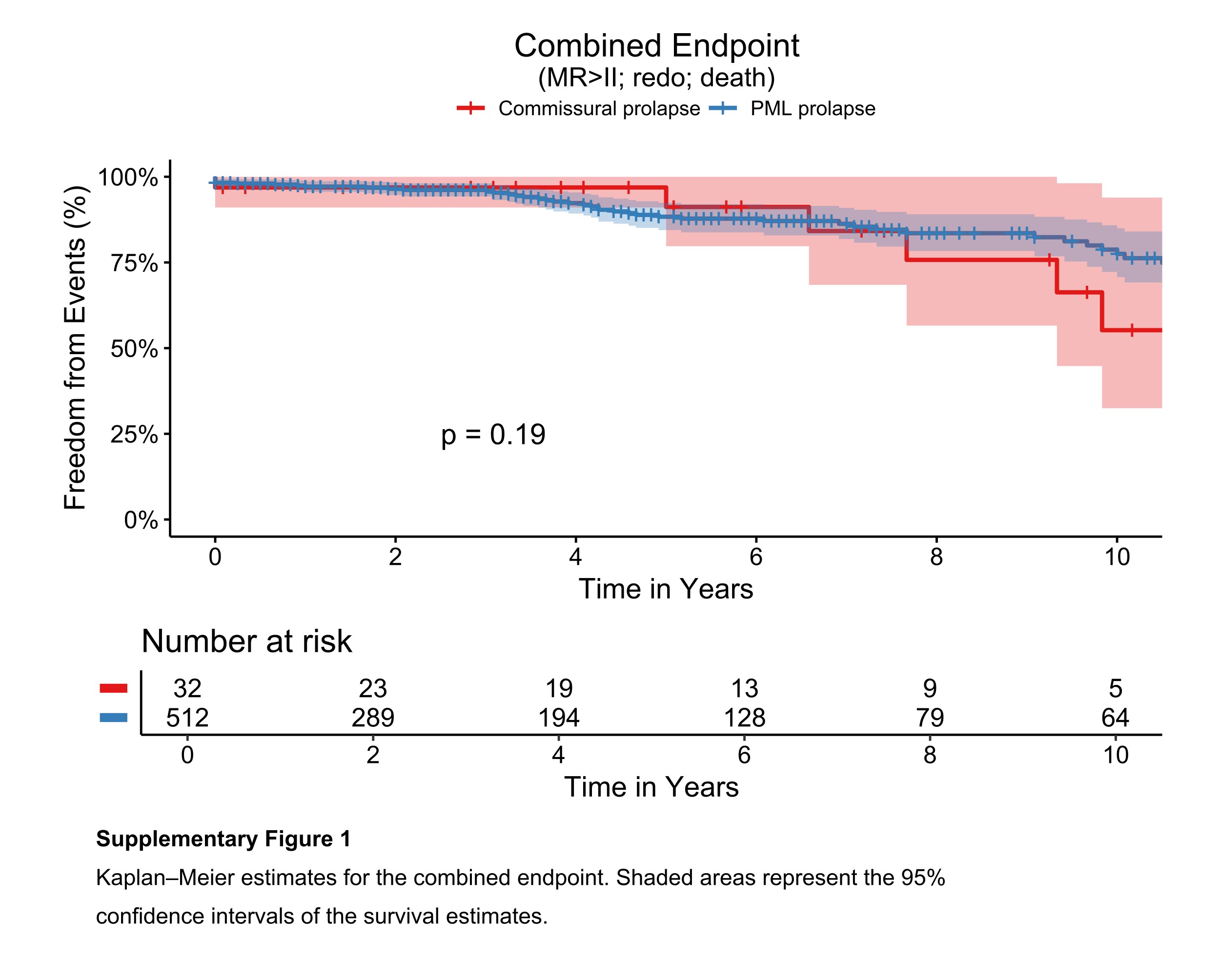

Supplement: ivaf213_Supplementary_Data [file ivaf213_supplementary_data.zip › Supplementary_Figure 1.jpg]

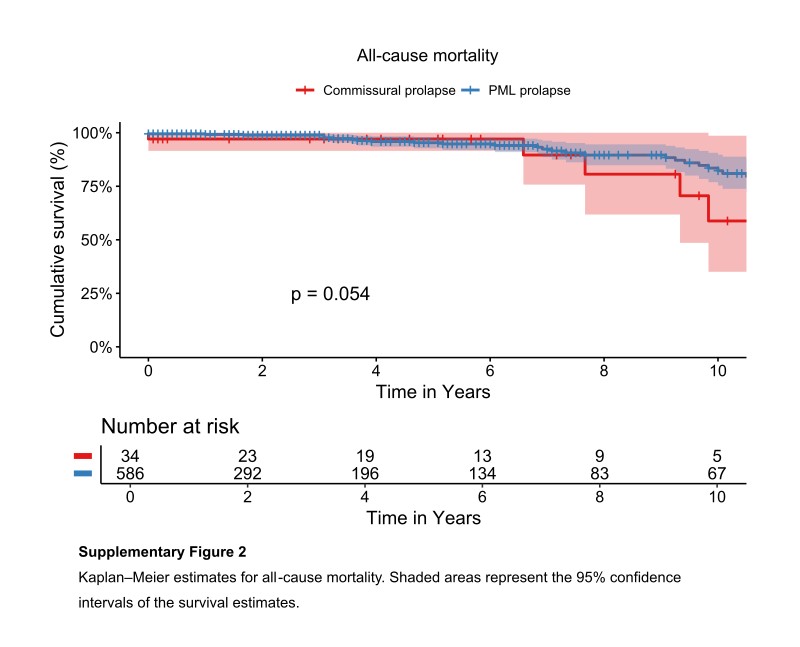

Supplement: ivaf213_Supplementary_Data [file ivaf213_supplementary_data.zip › Supplementary_Figure 2_all-cause.jpg]
